# Chemoresistance in Pancreatic Cancer: The Role of Adipose-Derived Mesenchymal Stem Cells and Key Resistance Genes

**DOI:** 10.3390/ijms26010390

**Published:** 2025-01-04

**Authors:** Shahram Parvaneh, Vanda Miklós, Zoltán Gábor Páhi, Diána Szűcs, Tamás Monostori, Szilárd Póliska, Viktória Venglovecz, Tibor Pankotai, Lajos Kemény, Zoltán Veréb

**Affiliations:** 1 Regenerative Medicine and Cellular Pharmacology Laboratory, Department of Dermatology and Allergology, University of Szeged, H-6720 Szeged, Hungary; shparvaneh79@gmail.com (S.P.); szucs.diana@med.u-szeged.hu (D.S.); monostori.tamas.bence@med.u-szeged.hu (T.M.); kemeny.lajos@med.u-szeged.hu (L.K.); 2 Doctoral School of Clinical Medicine, University of Szeged, H-6720 Szeged, Hungary; 3 Biobank, University of Szeged, H-6725 Szeged, Hungary; miklos.vanda96@gmail.com; 4 Genome Integrity and DNA Repair Core Group, Hungarian Centre of Excellence for Molecular Medicine (HCEMM), H-6728 Szeged, Hungary; pahizol@gmail.com (Z.G.P.); pankotai.tibor@szte.hu (T.P.); 5 Department of Pathology, Albert Szent-Györgyi Medical School, University of Szeged, H-6725 Szeged, Hungary; 6 Interdisciplinary Research Development and Innovation, Center of Excellence, University of Szeged, H-6720 Szeged, Hungary; 7 Genomic Medicine and Bioinformatics Core Facility, Department of Biochemistry and Molecular Biology, Faculty of Medicine, University of Debrecen, H-4032 Debrecen, Hungary; poliska@med.unideb.hu; 8 Department of Pharmacology and Pharmacotherapy, University of Szeged, H-6720 Szeged, Hungary; venglovecz.viktoria@med.u-szeged.hu; 9 HCEMM-SZTE Skin Research Group, University of Szeged, H-6720 Szeged, Hungary

**Keywords:** pancreatic cancer, Capan-1, adipose-derived mesenchymal stem cell (ASC), protein array, transcriptome, oxaliplatin, 5-fluorouracil

## Abstract

Drug resistance is a significant challenge in pancreatic ductal adenocarcinoma (PDAC), where stromal elements such as adipose-derived mesenchymal stem cells (ASCs) contribute to a chemoresistant tumor microenvironment (TME). This study explored the effects of oxaliplatin (OXP) and 5-fluorouracil (5-FU) on PDAC cells (Capan-1) and ASCs to investigate the mechanisms of chemoresistance. While OXP and 5-FU reduced Capan-1 viability in a dose- and time-dependent manner, ASCs demonstrated high resistance, maintaining > 90% viability even at cytotoxic doses. Transcriptomic analyses revealed OXP-induced transcriptional reprogramming in ASCs, with over 7000 differentially expressed genes, highlighting the pathways related to DNA damage response, cell cycle regulation, and stress-related signaling. In contrast, 5-FU elicited limited transcriptional changes, affecting only 192 genes. Cytokine proteome profiling revealed that OXP-treated ASCs significantly influenced the tumor microenvironment by promoting immune evasion (via IL-4, GM-CSF, IP-10, and GROα) and driving extracellular matrix remodeling (through EMMPRIN and DPPIV). In contrast, 5-FU induced comparatively weaker effects, primarily limited to hypoxia-related pathways. Although OXP reduced angiogenic factors, it paradoxically activated pro-survival pathways, thereby enhancing ASC-mediated tumor support. These findings underscore ASCs as modulators of chemoresistance via secretome alterations and stress adaptation. Therefore, future strategies should prioritize the precise targeting of tumor cells while also focusing on the development of personalized treatments to achieve durable therapeutic responses in PDAC.

## 1. Introduction

Pancreatic cancer is recognized as a highly aggressive human malignancy, often called a silent threat, because it typically shows no noticeable symptoms until advanced stages, making early diagnosis challenging and leading to poor patient outcomes [1,2]. Over the years, the incidence of pancreatic cancer has increased steadily [3]. The fatality rate within the first year of diagnosis is approximately 75%, with less than 10% survival rate over five years [3,4]. Pancreatic ductal adenocarcinoma (PDAC) represents 90% of all pancreatic tumors, and around 50% of PDAC patients have no clear symptoms in the early stages [5]. As a result, many patients are diagnosed at advanced stages, limiting the potential for radical surgery [5,6]. Consequently, patients must rely on chemotherapy and radiotherapy during later stages of treatment. This shift in the treatment strategies is influenced by the unique challenges posed by the tumor microenvironment (TME) in pancreatic cancer along with the intrinsic resistance of cancer cells to chemotherapy [7]. This resistance, in turn, allows specific malignant cells to survive, complicating treatment approaches even further [8]. Moreover, approximately 40% of patients with PDAC experience tumor recurrence even after surgical resection and die within a year [9]. Despite advancements in diagnostic techniques and the growing range of therapeutic options, the main challenge emerging after the latent phase of the disease stems from the tumor’s intrinsic resistance to chemotherapy [10]. However, most anticancer therapies are designed to target cancer cells, often neglecting the non-cancerous cells in the TME, which can profoundly influence the tumor’s response [11,12]. The TME consists of several components, including the extracellular matrix, endothelial cells, immune cells, cancer-associated fibroblasts (CAFs), and mesenchymal stromal cells (MSCs) [13]. Cancer cells secrete chemotactic signals that recruit adipose-derived stem cells (ASCs) from local tissues and MSCs from the bone marrow [14]. These recruited cells can differentiate into tumor-associated MSCs (TAMSCs) or CAFs [15]. These stromal cells play an essential role in constructing the connective tissue and extracellular matrix within the tumor, and their contribution to chemotherapy resistance is a crucial area of investigation [15,16]. Numerous studies have demonstrated that interactions between MSCs and tumor cells shape the TME and influence cancer progression in various malignancies, including breast, gastric, pancreatic, and colorectal cancers [17,18,19,20]. A key mechanism under investigation is ASC-mediated mitochondrial transfer, which supports cancer cell survival and metabolic adaptation, contributing to the emergence of resistance to therapies [21,22]. In PDAC, MSC recruitment to the TME is linked with disease progression and therapy resistance [23]. Bone marrow-derived MSCs, for example, differentiate into CAFs or TAMSCs in PDAC tumors, contributing to the CAF population [24]. Adipose tissue is another critical source of CAFs in PDAC due to the pancreas’s retroperitoneal location near adipose deposits [25,26]. ASCs can differentiate into two distinct CAF subpopulations: myoblastic CAFs (myCAFs) or inflammatory CAFs (iCAFs), depending on their proximity to PDAC cells in vitro [27]. The interaction between stromal cells and tumor cells is regulated by extracellular vesicles, cytokines, chemokines, and direct cell-to-cell communication, all promoting CAF activation and tumor growth [28]. MSCs are known to secrete a wide array of cytokines that play key roles in angiogenesis, ECM remodeling, and immune modulation, all crucial in supporting cancer progression and metastasis [29]. Several intracellular pathways, including JAK/STAT, mTOR, and NF-κB, mediate the stromal–tumor crosstalk [30,31,32]. Furthermore, MSCs have been investigated as potential carriers for anticancer therapies because of their homing ability into tumor sites [33]. While their well-known properties such as self-renewal, immune regulation, and differentiation capacity are well established, their role in contributing to tumor chemoresistance is still not fully understood [34]. For example, bone marrow-derived MSCs have been shown to enhance the chemoresistance of CD133-expressing gastric cancer cells via activation of the PI3K/Akt pathway, increasing Bcl-2 expression and decreasing BAX levels [35]. Oxaliplatin (OXP) and 5-fluorouracil (5-FU) are two chemotherapy agents commonly used, either alone or in combination with other regimens, for PDAC treatment [36,37]. 5-FU inhibits thymidylate synthase (TS) and incorporates its metabolites into RNA and DNA, while OXP, a platinum-based drug, interferes with DNA replication by forming DNA adducts, leading to cell death [38,39,40].

This study investigates the effects of the chemotherapeutic agents 5-FU and OXP on the PDAC cell line (Capan-1) and ASCs. We aim to evaluate the cytotoxic impact of these drugs on both cancerous and stromal cells. Furthermore, we perform transcriptomic and protein array analyses of ASCs following chemotherapy treatment to uncover changes in the gene expression and cytokine secretion profile that may illuminate the interactions between stromal cells and the mechanisms underlying chemoresistance. These findings provide valuable insights into the molecular alterations in ASCs induced by 5-FU and OXP, advancing our understanding of the role of stromal cells in chemoresistance in PDAC.

## 2. Results

### 2.1. Analysis of OXP and 5-FU Cytotoxicity on Capan-1 Cells and ASCs

**Figure 1:**
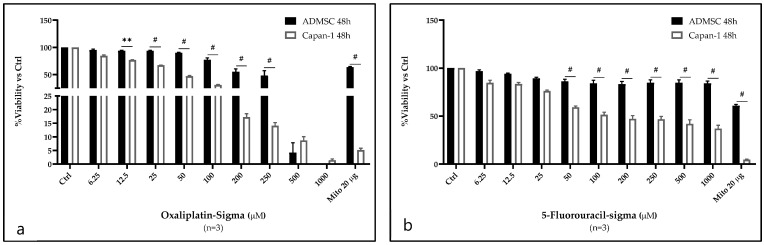
Evaluating the cytotoxic effects of OXP and 5-FU on Capan-1 cells and ASCs using MTT assay. The cells were treated for 48 h with concentrations (ranging from 6.25 to 1000 μM) of (**a**) OXA and (**b**) 5-FU. Cell viability is expressed as a percentage of untreated control (Ctrl). Mitomycin (Mito) 20 μg/mL was used as the positive control. (mean ± SD, *n* = 3). ** *p* < 0.01 and # *p* < 0.0001.

Using the MTT assay, the cytotoxicity effects of OXP and 5-FU were evaluated on the Capan-1 cell line at 24 and 48 h (Figure S1) and on ASCs at 48 h. The results showed a significant decrease in cell viability as the concentration of the anticancer drugs increased (ranging from 6.25 to 1000 μM), indicating a dose- and time-dependent pattern in response. Higher concentrations led to more severe effects on cell viability. For both Capan-1 and ASCs, OXP exhibited the highest cytotoxicity effects (Figure 1a,b, and Table S1).

The half-maximal inhibitory concentration (IC50) values, representing the concentration at which 50% of cell growth is inhibited, are reported in Table S1. The effects of OXP and 5-FU on Capan-1 cells at 24 and 48 h were statistically significant compared with untreated controls at concentrations of 50 µM or higher. While the IC50 for 5-FU was not reached at 24 h, even at the highest concentration of 1000 μM (Figure S1), the IC50 for OXP was successfully achieved within this treatment time.

The MTT assay results evaluating the cytotoxic effects of anticancer treatments on ASCs revealed an IC50 value for OXP only at concentrations of 250 μM and higher (Figure 1a,b and Table S1). Remarkably, at a concentration of 50 μM, equivalent to the IC50 for Capan-1 cells, the viability of ASCs remained above 90% at 48 h post-treatment. Moreover, even 48 h after treatment with 1000 μM of 5-FU, the viability of ASCs consistently remained above 90% (Figure 1a,b and Table S1). The comparison of the antiproliferative effects of 5-FU (Accord), administered to patients in the hospital, on Capan-1 and ASC cells after 48 h of treatment revealed a similar pattern to that we used (Sigma) in all our experiments (Figure S1D). Even after 48 h of treatment at concentrations of 50 µM or higher of OXP and 5-FU, ASCs exhibited more resistance to these anticancer drugs than Capan-1 cells. Notably, for the mitomycin (Mito), which was used as the positive control, the IC50 was reached in Capan-1 cells at 24 h post-treatment (Figure S1). In contrast, even after 48 h of treatment with Mito in ASCs, the IC50 was not reached, highlighting the high resistance of ASCs to cytotoxic treatment (Figure 1a,b).

### 2.2. OXP Treatment-Induced Pronounced Gene Expression Changes Compared with 5-FU Treatment and Untreated Controls in ASCs

**Figure 2:**
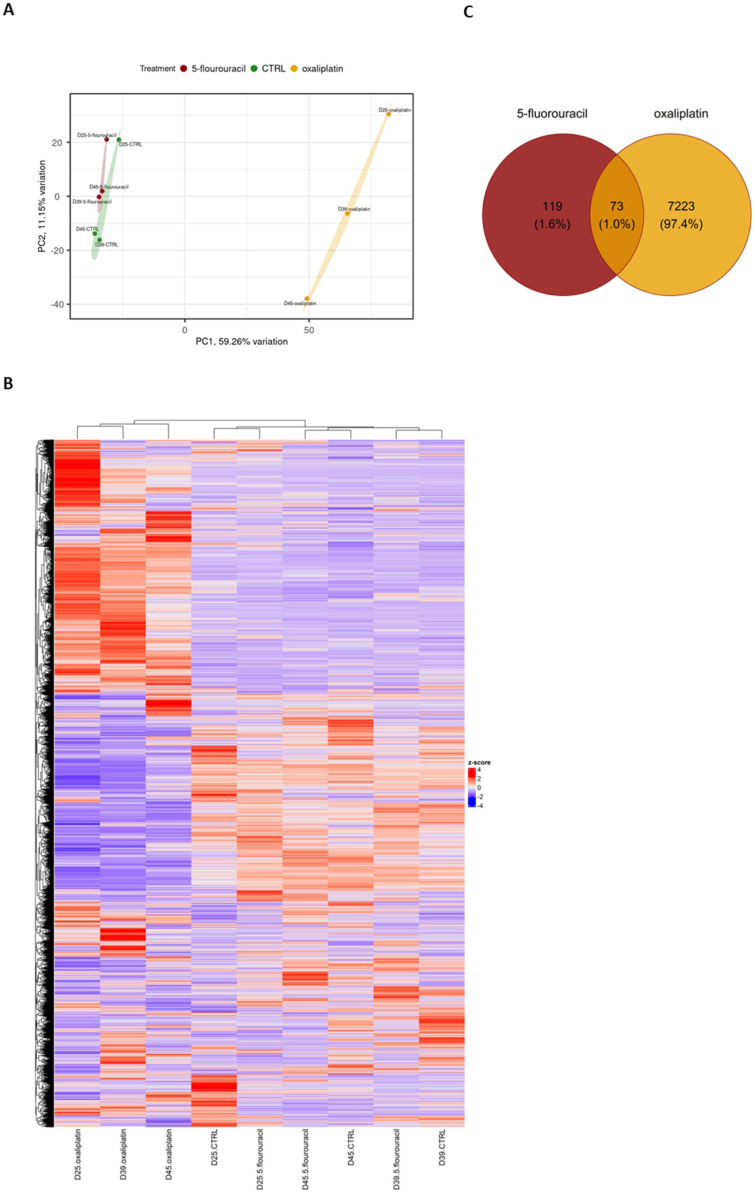
The overall results of RNA sequencing analysis comparing 5-FU and OXP-treated ASCs with the untreated control are presented in (**A**) PCA plot, (**B**) heatmap, and (**C**) Venn diagrams. D25, D39, and D45 represent three biological donor codes (*n* = 3).

RNA sequencing of ASCs treated with OXP and 5-FU revealed significant differences between the OXP-treated samples compared with the 5-FU-treated and untreated samples. Interestingly, while there were no significant differences between the untreated controls and the 5-FU-treated samples, the OXP treatment induced substantial alterations in gene expression. In the principal component analysis (PCA), ASC samples treated with 5-FU treatments were clustered with the untreated controls, indicating similar gene expression patterns. In contrast, the OXP-treated samples formed a distinct cluster, suggesting a unique transcriptional response (Figure 2A). Specifically, the analysis showed that 7296 genes were differentially expressed in response to OXP treatment compared with the untreated controls, whereas only 192 differentially expressed genes (DEGs) were found in response to 5-FU treatment. Moreover, 73 DEGs were identified to be commonly altered by both treatments. In the OXP treatment group, among the DEGs, 4043 genes were upregulated, and 3253 were downregulated compared with the untreated controls (Figure 2C).

**Figure 3:**
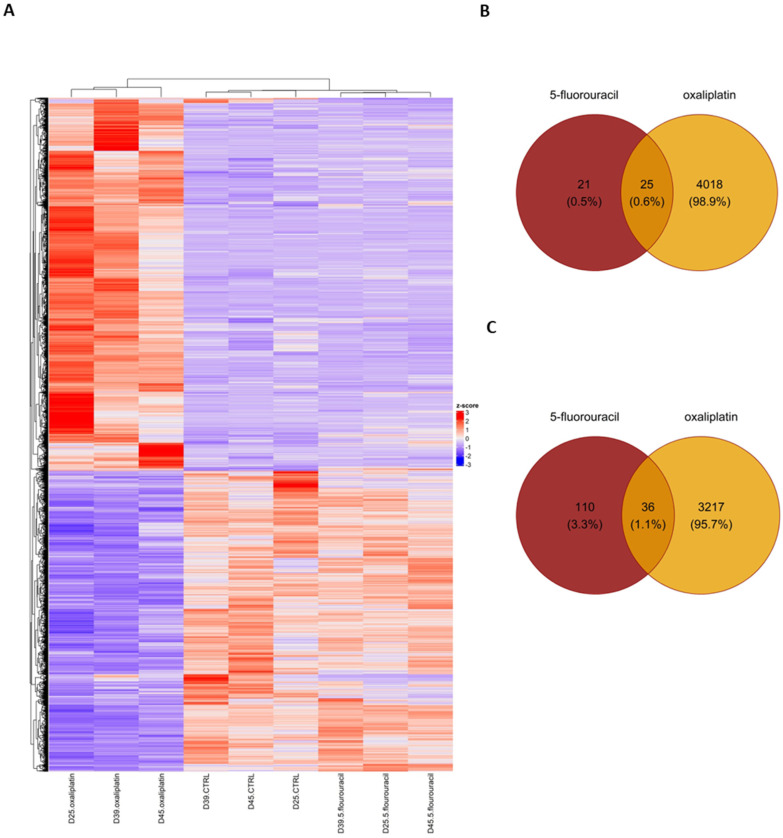
Significantly differentially expressed genes (DEGs) in ASCs treated with 5-FU and OXP compared with untreated controls. (**A**) The heatmap shows distinct clustering of control, 5-fluorouracil, and oxaliplatin treatments when only differentially expressed genes (DEGs) are plotted. Venn diagrams of (**B**) upregulated genes and (**C**) downregulated genes. D25, D39, and D45 represent three different biological donor codes (*n* = 3).

Within the DEGs identified in the 5-FU group, 46 genes were upregulated and 146 genes were downregulated compared with untreated controls (Figure 3C).

### 2.3. Gene Ontology (GO) Enrichment Analysis of DEGs in ASCs Treated with OXP and 5-FU

We performed a comprehensive GO enrichment analysis on the DEGs identified in the ASCs treated with OXP and 5-FU. The study revealed several significant enrichments in biological processes (BPs). Among the positively enriched GO terms, several crucial pathways involved in cellular responses to various stimuli and checkpoints in cell-cycle progression were identified. One notable enrichment is associated with the GO term “GO:0006977”, which involves “DNA damage response, signal transduction by p53 class mediator resulting in cell cycle arrest”. This GO term enrichment suggests increased activity in pathways responsible for detecting DNA damage and triggering cell cycle arrest mediated by p53 class proteins. Genes such as MDM2 and PIDD1 are implicated in this process (Table 1).

Among the positively enriched GO terms, several biological processes are notably represented, including the negative regulation of cell cycle progression (GO:0010948), DNA damage response (GO:0006977), cellular response to hypoxia (GO:0071456), and mitotic G1 DNA damage checkpoint signaling (GO:0031571) (Table 2). These findings indicate active mechanisms controlling cell cycle progression, responses to environmental stresses such as hypoxia, and the maintenance of genome stability. Moreover, the enrichment analysis highlights the involvement of specific genes in these processes, including B-cell Lymphoma 6 (*BCL6*), *MDM2*, *PIDD1*, and *RBM14*, among others (Table 1.) Among the negatively enriched GO terms for 5-FU and OXP, we identified exciting candidates, including Aurora kinase A (AURKA), B-cell Lymphoma 6 (BCL6), and breast cancer gene 1 (BRCA1) (Table 1). The expression patterns of genes that determine the biological pathways involved in tumorigenesis clearly show that oxaliplatin causes marked changes, whereas 5-FU is more like the untreated control, showing that it does not affect mesenchymal stem cells as it does tumor cells (Figures S2 and S3).

### 2.4. Differentially Expressed Gene Set Enrichment (GSEA) Analysis Results for ASCs Treated with Oxaliplatin

**Figure 4:**
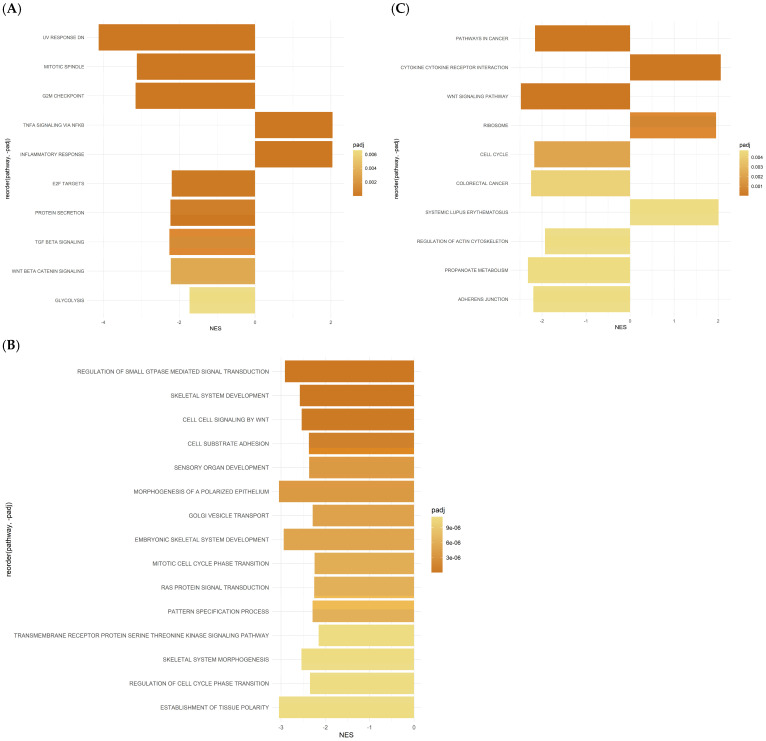
Differentially expressed gene set enrichment analysis (GSEA) results for ASCs treated with OXP: (**A**) Hallmark gene sets, (**B**) KEGG pathways, and (**C**) GO term gene sets pathways.

Pathway analysis revealed the dysregulation of several BPs in response to OXP treatment. Notably, analysis of the hallmark gene set pathways following OXP treatment revealed several pathways exhibiting significant regulation, each associated with diverse BPs, as shown in Figure 4A and Table 2. Among these, the HALLMARK_E2F_TARGETS pathway, with a significant *p*-value of 2.64 × 10^−5^ and a normalized enrichment score (NES) of −2.19, plays a crucial role in regulating the transcriptional activity of E2F transcription factors, which comprise 66 genes.

Similarly, the HALLMARK_PROTEIN_SECRETION pathway showed significant enrichment (*p*-value: 5.92 × 10^−5^, NES: −2.23), suggesting alterations in protein secretion mechanisms. This pathway consists of 48 genes crucial to intracellular trafficking and cellular communication. Furthermore, the HALLMARKs_TGF_BETA_SIGNALING and WNT_BETA_CATENIN_SIGNALING pathways displayed significant enrichment, with *p*-value: 0.00022, NES: −2.26 and *p*-value: 0.00059, NES: −2.22, respectively. These pathways are implicated in cell signaling and development. Another important pathway, HALLMARK_GLYCOLYSIS, demonstrated significant enrichment, with a *p*-value of 0.00132 and an NES of −1.73. This pathway, comprising 71 genes, is central to cellular energy metabolism, particularly glycolytic processes. Additionally, the HALLMARK_APICAL_SURFACE and HALLMARK_ANDROGEN_RESPONSE pathways were identified as significantly enriched (*p*-value: 0.00178, NES: −2.04 and *p*-value: 0.00268, NES: −1.77, respectively). These pathways are associated with epithelial cell polarity and androgen-mediated signaling events. Moreover, the HALLMARKs_IL6_JAK_STAT3_SIGNALING and INTERFERON_GAMMA_RESPONSE pathways showed significance with *p*-values of 0.00835 and 0.01327, respectively (Figure 4A, Table 2). These pathways play crucial roles in the immune response modulation and cytokine signaling pathways.

**Figure 5:**
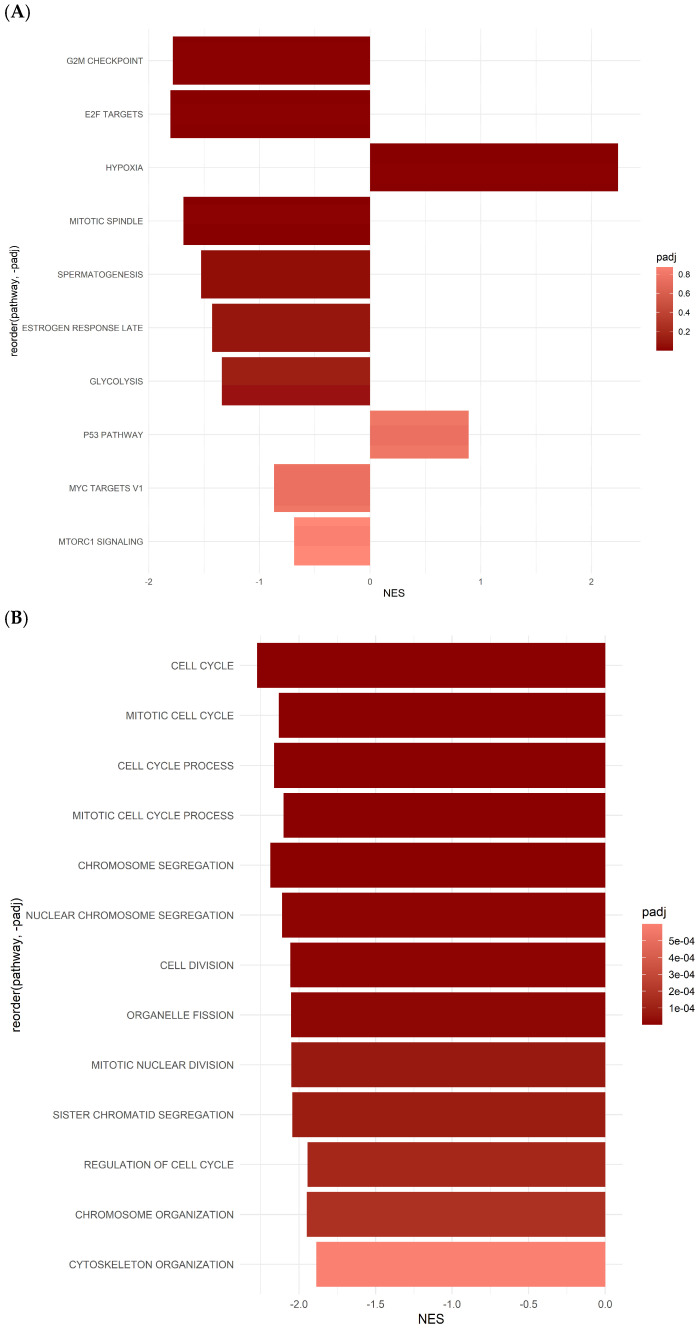
GSEA results from the Hallmark and GO Pathway databases for ASCs treated with 5-FU. (**A**) Hallmark gene sets, (**B**) GO term gene sets.

The exploration through KEGG (Kyoto Encyclopedia of Genes and Genomes) annotations uncovers diverse enriched pathways, highlighting their roles in biological processes (Figure 4B and Table 3). Sitting prominently within the top 10 is the KEGG_CYTOKINE_CYTOKINE_RECEPTOR_INTERACTION pathway, boasting a strikingly low p-value of 3.94 × 10^−7^ and NES of 2.06, indicating its positive significant enrichment. This pathway is essential for intercellular communication, primarily through cytokine–receptor interactions. Another important pathway is the KEGG_PATHWAYS_IN_CANCER, which demonstrates considerable enrichment with a *p*-value of 4.23 × 10^−7^ and NES of −2.16, suggesting a potential downregulation. This pathway remains central to cancer biology, offering critical insights into tumor progression and malignancy. Another standout contender is the KEGG_WNT_SIGNALING_PATHWAY, boasting a *p*-value of 7.93 × 10^−7^. Despite its negative NES of −2.48, suggesting downregulation, this pathway remains pivotal to developmental processes and disease pathology, notably cancer. In contrast, the KEGG_RIBOSOME pathway, while exhibiting a higher *p*-value of 1.80 × 10^−5^, highlights a positive NES of 1.95. This pathway is the cornerstone of protein synthesis and ribosomal function, vital for maintaining cellular equilibrium. Further, the KEGG_CELL_CYCLE pathway hints at potential downregulation with a *p*-value of 6.57 × 10^−5^ and a negative NES of −2.17. Orchestrating the orderly progression of cell division, this pathway underscores its importance in regulating cellular proliferation and growth, which the heatmaps of DEGS showed in Figure 5. Moreover, pathways like KEGG_COLORECTAL_CANCER and KEGG_ADHERENS_JUNCTION exhibit notable enrichment, suggesting their roles in colorectal cancer development and cell–cell adhesion processes. The analysis extends to metabolic pathways, such as KEGG_PROPANOATE_METABOLISM, hinting at potential dysregulation in metabolic processes. KEGG_SYSTEMIC_LUPUS_ERYTHEMATOSUS emerges, emphasizing its link to autoimmune disorders (Figure 4B, Table 2). According to the KEGG analysis, colorectal, endometrial, basal cell carcinoma, and pancreatic cancer-associated pathways were activated (Figures S2 and S3).

The following results are from Gene Ontology (GO) analysis, which categorizes genes based on their functions in biological processes (BPs), molecular functions (MFs), and cellular components (CCs) (Figure 4B, Table 2). In the GOBP_REGULATION_OF_SMALL_GTPASE_MEDIATED_SIGNAL_TRANSDUCTION pathway, with a notably low *p*-value of 5.10 × 10^−13^ and a negative normalized enrichment score (NES) of −2.91, there is a suggestion of potential downregulation. This pathway is crucial for regulating small GTPase-mediated signal transduction, which is vital for cell growth, differentiation, and migration. The GOMF_CYTOKINE_ACTIVITY pathway, which has a *p*-value of 7.15 × 10^−13^ and an NES of 2.56, indicates significant cytokine activity enrichment. This underscores its importance in modulating immune responses, inflammation, and cellular communication (Figure 4C, Table 2).

Similarly, the pathway GOMF_SIGNALING_RECEPTOR_REGULATOR_ACTIVITY, with a *p*-value of 5.38 × 10^−13^ and an NES of 2.30, emphasizes the role of signaling receptor regulator activity. It fine-tunes signal transduction cascades, ensuring cellular balance. In the case of GOBP_SKELETAL_SYSTEM_DEVELOPMENT, boasting an enrichment *p*-value of 8.64 × 10^−12^ and an NES of −2.58, it highlights its involvement in skeletal system development, crucial for tissue formation and maintenance. The pathway GOMF_GTPASE_ACTIVATOR_ACTIVITY, exhibiting a *p*-value of 1.01 × 10^−10^ and an NES of −2.68, indicates significant enrichment in GTPase activator activity, crucial for intracellular signaling and vesicle trafficking.

Similarly, for GOMF_TUBULIN_BINDING, the *p*-value of 1.21 × 10^−10^ and NES of −2.52 signify substantial enrichment in tubulin binding, essential for cell division and intracellular transport. Regarding GOBP_CELL_CELL_SIGNALING_BY_WNT, featuring a *p*-value of 4.04 × 10^−10^ and an NES of −2.54, it suggests involvement in WNT signaling-mediated cell–cell communication, critical for developmental processes. Exhibiting a *p*-value of 6.23 × 10^−10^ and an NES of −2.48, GOCC_GOLGI_APPARATUS_SUBCOMPARTMENT indicates enrichment in the Golgi apparatus sub-compartment, pivotal for protein modification and trafficking. Moreover, GOMF_PHOSPHORIC_ESTER_HYDROLASE_ACTIVITY, boasting a *p*-value of 1.31 × 10^−9^ and an NES of −2.42, suggests significant enrichment in phosphoric ester hydrolase activity, crucial for cellular metabolism and signaling. Finally, the pathway GOMF_CYTOKINE_RECEPTOR_BINDING, with a *p*-value of 1.51 × 10^−9^ and an NES of 2.25, highlights enrichment in cytokine receptor binding, indicating its role in immune response regulation (Figure 4C, Table 2).

### 2.5. Gene Set Enrichment Analysis (GSEA) Reveals Hallmark and GO Pathways in ASCs Treated with 5-FU

Several hallmark gene pathways exhibit notable responses in 5-FU-treated ASCs (Figure 5A and Figure S2, Table 3). The G2M_CHECKPOINT pathway, with a *p*-value of 0.0000733 and a significant NES of −1.78, indicates a potential downregulation of the genes associated with the G2/M checkpoint regulation, critical for cell cycle progression. Similarly, the E2F_TARGETS pathway, showing a *p*-value of 0.000136 and an NES of −1.81, suggests alterations in genes regulated by E2F transcription factors, indicative of potential cycle dysregulation.

In contrast, the MITOTIC_SPINDLE pathway, exhibiting a *p*-value of 0.00196 and an NES of −1.69, suggests potential downregulation of the genes involved in mitotic spindle assembly and function, essential for proper cell division. The SPERMATOGENESIS pathway, with a *p*-value of 0.0239 and an NES of −1.53, indicates potential gene alterations associated with spermatogenesis processes. Furthermore, the ESTROGEN_RESPONSE_LATE pathway, displaying a *p*-value of 0.0531 and an NES of −1.43, suggests potential downregulation of the genes regulated by estrogen signaling, which may affect late-stage estrogen responses. The GLYCOLYSIS pathway, with a *p*-value of 0.100 and an NES of −1.34, indicates potential alterations in the glycolytic pathway genes crucial for energy metabolism. Conversely, the HYPOXIA pathway (*p*-value = 0.000934, NES = 2.24) was significantly upregulated under 5-FU treatment (Figure 5A, Table 3). This pathway plays an essential role in controlling various cellular response signaling cascades.

Notably, the P53_PATHWAY and MYC_TARGETS_V1 pathways show non-significant responses, with *p*-values of 0.605 and 0.676, respectively. These pathways may not exhibit substantial changes in gene expression under 5-FU treatment in ASCs. Similarly, the MTORC1_SIGNALING pathway, with a *p*-value of 0.874, suggests minimal enrichment, indicating limited involvement in the cellular response to fluorouracil treatment (Figure 5A, Table 3).

The GO analysis of cells treated with 5-FU shows significant changes in various BPs and CCs gene sets. Several GO terms associated with cell cycle regulation exhibit pronounced dysregulation following 5-FU treatment. For instance, terms such as “GOBP_CELL_CYCLE”, “GOBP_CELL_CYCLE_PROCESS”, “GOBP_CELL_DIVISION”, and “GOBP_MITOTIC_CELL_CYCLE” show significant *p*-values, indicating their involvement in response to 5-FU-induced stress. These findings suggest that fluorouracil treatment disrupts normal cell cycle progression, inducing cell cycle arrest or aberrant cell division (Figure 5B, Table 3).

Moreover, GO terms associated with chromosome organization and segregation, such as “GOBP_CHROMOSOME_ORGANIZATION”, “GOBP_CHROMOSOME_SEGREGATION”, and “GOBP_NUCLEAR_CHROMOSOME_SEGREGATION”, are also significantly affected. This implies that 5-FU treatment may interfere with the proper organization and segregation of chromosomes during cell division, potentially leading to genomic instability and cell death. Additionally, alterations in cytoskeleton organization-related terms, including “GOBP_CYTOSKELETON_ORGANIZATION” and “GOCC_MICROTUBULE_CYTOSKELETON”, suggest perturbations in the cytoskeletal dynamics induced by 5-FU. Disruption of the cytoskeleton could affect various cellular processes, including cell shape maintenance, intracellular transport, and cell division. Furthermore, GO terms related to organelle dynamics, such as “GOBP_ORGANELLE_FISSION”, highlight the potential impacts of 5-FU on organelle integrity and dynamics within the cell. The GO analysis indicates that fluorouracil treatment profoundly affects fundamental cellular processes, particularly cell cycle regulation, chromosome dynamics, cytoskeletal organization, and organelle dynamics (Figure 5B, Table 3).

### 2.6. Protein Array Analysis of ASCs Supernatant Treated with OXP and 5-FU

**Figure 6:**
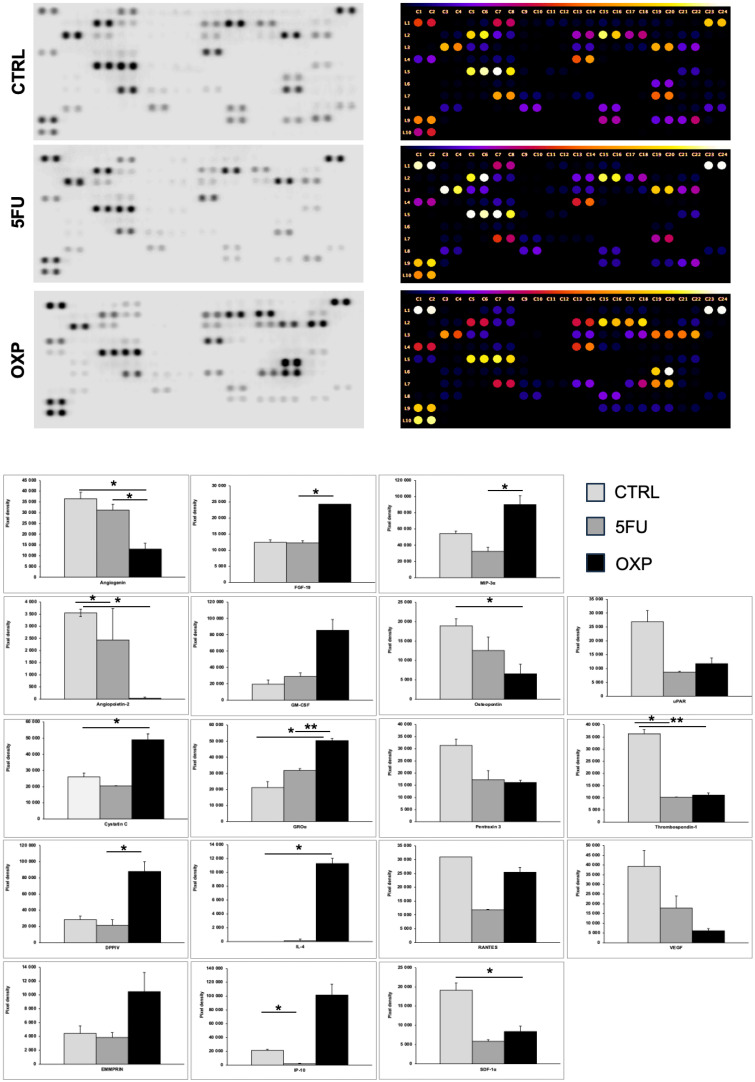
Cytokine expression profile of ASCs following 5-FU and OXP treatment. Compared with untreated controls, the cytokine secretion profile of ASCs at 48 h post-treatment were assessed using the proteome profiler human cytokine array (mean ± SD, *n* = 3). **FGF-19**: Fibroblast Growth Factor 19, **GM-CSF**: Granulocyte–Macrophage Colony-Stimulating Factor, **GROα**: Growth-Regulated Oncogene Alpha (CXCL1), **IL-4**: Interleukin 4, **IP-10**: Interferon Gamma-Induced Protein 10 (CXCL10), **DPPIV**: Dipeptidyl Peptidase IV (CD26), **MIP-α**: Macrophage Inflammatory Protein-alpha (CCL3), **SDF-1α**: Stromal Cell-Derived Factor 1 (CXCL12), **uPAR**: Urokinase Plasminogen Activator Receptor, **VEGF**: Vascular Endothelial Growth Factor. Although twofold changes were measured for several cytokines, the significance values ranged from *p* = 0.05 to *p* = 0.06. (* *p* ≤ 0.05, ** *p* ≤ 0.01,).

A large-scale protein array was conducted to assess the cytokine expression profiles of ASCs treated with OXP and 5-FU, compared with untreated controls (Figure 6). Both treatments markedly reduced pro-angiogenic factors, including angiogenin, angiopoietin-2, and VEGF, with OXP inducing a more pronounced anti-angiogenic effect. In contrast, stress-related markers such as cystatin C and extracellular matrix modulators like EMMPRIN were significantly upregulated under OXP treatment, while 5-FU had minimal impact. Immune-modulatory and pro-inflammatory factors showed divergent responses, with OXP causing a substantial increase in GM-CSF, GROα, FGF-19, and IL-4 as well as a significant elevation in IP-10 and MIP-3α, whereas 5-FU demonstrated a moderate or suppressive effect on these factors. Osteopontin expression was consistently suppressed by both treatments, while pentraxin 3 and RANTES levels decreased, although OXP partially restored RANTES levels compared with 5-FU. Additionally, SDF-1α and thrombospondin-1 were reduced by both treatments, but OXP slightly increased uPAR expression relative to 5-FU. These results reveal distinct and overlapping effects of OXP and 5-FU on the ASC secretome, highlighting their roles in modulating angiogenesis, inflammation, and extracellular matrix remodeling.

## 3. Discussion

MSCs have been explored as potential carriers for anticancer therapies due to their tumor-homing capabilities [33]. Recently, increasing attention has been given to the dynamic interactions between tumor and stromal cells in promoting cancer drug resistance [41,42]. Stromal cells, which comprise a massive portion of the TME’s cellular elements, influence tumor metabolism, growth, metastasis, immune evasion, and treatment resistance [28,42,43]. Additionally, it has been observed that ASCs can differentiate into cells that contribute to tumor cell invasion and survival via several mechanisms in laboratory settings [21,22,25,26].

This study offers important insights into the cytotoxic effects of OXP and 5-FU on PDAC cells (Capan-1) and ASCs, emphasizing both the potential and the challenges of these treatments. Furthermore, transcriptomic analyses and the cytokine secretion profile of ASCs treated with OXP and 5-FU revealed significant alterations in gene expression and cytokine release, shedding light on the potential complex interactions between stromal cells and the mechanisms underlying chemoresistance.

The MTT assay results reveal a striking difference in how Capan-1 cells and ASCs respond to the anticancer drugs. As expected, the Capan-1 cells show a time- and dose-dependent reduction in viability, with OXP exhibiting the highest cytotoxicity. In contrast, the high resistance of ASCs to these treatments is notable. Even at cytotoxic concentrations to Capan-1 cells (e.g., 50 μM of OXP), ASCs maintain viability above 90%, even after 48 h of treatment. This suggests that ASCs possess intrinsic protective mechanisms, possibly related to their stem cell-like properties, such as efficient DNA repair systems, lower proliferative rates, or increased resistance to apoptosis [43]. Noting that ASCs exhibited higher viability than Capan-1 cells across drug treatments, we focused our investigation on the efficacy of 5-FU and OXP to explore resistance mechanisms in ASCs at both the transcriptomic and protein array levels. The gene expression data reveal stark differences between OXP and 5-FU in their effects on ASCs’ gene regulation, with OXP showing profound transcriptional changes, while 5-FU has minimal impact. The significant transcriptional reprogramming caused by OXP highlights its broad effects on cellular processes in ASCs. Over 7000 genes are differentially expressed in response to OXP treatment, with many linked to critical biological processes such as DNA damage response, cell cycle regulation, and cellular stress responses (e.g., p53-mediated pathways, centriole replication, and cytokine signaling responses). The enrichment of GO terms related to “DNA damage response, signal transduction by p53 class mediator resulting in cell cycle arrest” underscores OXP’s role in inducing cell cycle arrest through DNA damage, which aligns with its known mechanism of action. This suggests that OXP effectively triggers apoptotic pathways or senescence, particularly in fast-dividing cells like cancer cells, but it might induce similar stress responses in ASCs at higher concentrations. Additionally, the GSEA analysis shows that hallmark pathways such as E2F targets, protein secretion, TGF-beta signaling, and Wnt/Beta-catenin signaling are significantly altered by OXP. These pathways are vital for cell proliferation, survival, and communication, and their dysregulation by OXP could contribute to both its therapeutic efficacy in cancer and its potential off-target side effects.

In contrast, 5-FU has a limited impact on ASCs, with only 192 differentially expressed genes, suggesting that ASCs exhibit minimal changes in gene expression in response to 5-FU, with profiles like the untreated controls. The GO and GSEA analyses reveal that while 5-FU induces some changes in pathways related to hypoxia, cell cycle, and chromosome organization, these effects are far less pronounced than those caused by OXP. This finding is promising, as it suggests that 5-FU may exert a more selective cytotoxic effect on cancer cells, while sparing normal stromal cells and potentially minimizing off-target toxicity.

**Figure 7:**
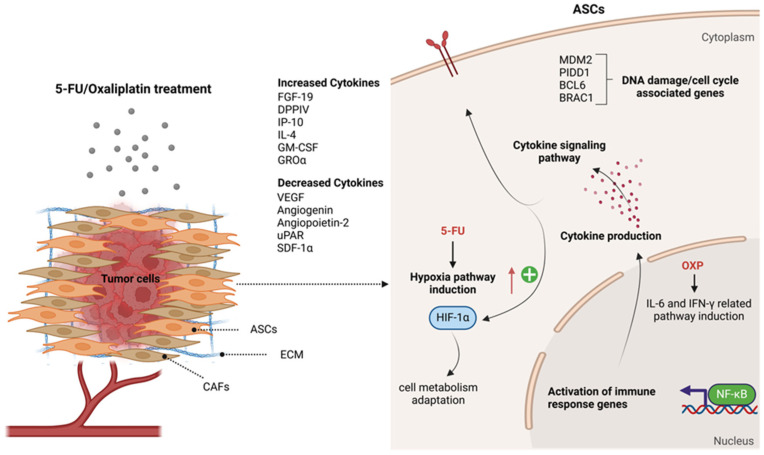
Schematic illustration of the effects of 5-FU and OXP treatments on ASCs, their potential role in the PDAC tumor microenvironment, and the development of chemotherapy resistance. **CAFs**: cancer-associated fibroblasts, **ECM**: extracellular matrix, **FGF-19**: Fibroblast Growth Factor 19, **DPPIV**: Dipeptidyl Peptidase IV (CD26), **IP-10**: Interferon Gamma-Induced Protein 10 (CXCL10), **IL-4**: Interleukin 4, **GM-CSF**: Granulocyte–Macrophage Colony-Stimulating Factor, **GROα**: Growth-Regulated Oncogene Alpha (CXCL1), **VEGF**: Vascular Endothelial Growth Factor, **uPAR**: Urokinase Plasminogen Activator Receptor, **SDF-1α**: Stromal Cell-Derived Factor 1 (CXCL12), **HIF-1α**: Hypoxia-Inducible Factor 1, **IL-6**: Interleukin 6, **IFNγ**: Interferon Gamma, **NF-κB**: Nuclear Factor Kappa B. Created in BioRender.com.

The GO enrichment analysis of the DEGs in ASCs treated with OXP and 5-FU revealed significant enrichment in biological processes related to cellular responses to hypoxia, DNA damage response (DDR), and cell cycle regulation. Genes involved in these processes, such as *MDM2*, *PIDD1*, and *RBM14*, play crucial roles in orchestrating cellular responses to various stimuli and DNA damage [44]. We observed enrichment in the DDR pathway mediated by p53 class proteins, suggesting increased activity in detecting DNA damage and initiating cell cycle arrest [45]. Recent studies have emphasized the critical balance between MDM2 and p53 in the maintenance of ASCs and the viability of hematopoietic stem cells [46]. Among the DEGs identified in ASCs treated with OXP and 5-FU, Aurora kinase A (AURKA), B-cell Lymphoma 6 (BCL6), and breast cancer gene 1 (BRCA1), were also found to play essential roles in regulating the DNA damage response and cell cycle processes in ASCs (Figure 7).

BCL6, a transcriptional repressor linked to blood cancers and solid tumors, suppresses tumor suppressor genes while promoting those involved in cell proliferation and immune evasion, contributing to chemotherapy resistance [47,48,49]. Targeting BCL6 has increased cancer cell sensitivity to chemotherapy, suggesting its inhibition could overcome resistance in solid tumors [50,51]. The dysregulated BCL6 expression in our study may similarly promote chemoresistance in ASCs. BRCA1, crucial for DNA repair, showed increased expression in our transcriptome analysis, suggesting potential enhanced DNA repair mechanisms that reduce the genotoxic effects of 5-FU and OXP, aiding cell survival [52]. Pathway analysis also revealed the downregulation of the G2/M checkpoint and E2F target pathways, which may impair cell cycle arrest and promote genomic instability [53]. Activation of the IL6/JAK/STAT3 and IFN-γ signaling pathways highlights the role of immune modulation in chemoresistance, consistent with the cytokine array results and the well-established function of NF-κB/STAT3 in upregulating anti-apoptotic proteins and driving resistance to 5-FU and OXP [54,55]. Targeting the NF-κB/STAT3 pathway could therefore provide a potential strategy to overcome chemoresistance in pancreatic cancer treatment.

The upregulation of the hypoxia signaling pathway in 5-FU-treated ASCs indicates an adaptive response to chemotherapy-induced stress, aimed at compensating for disrupted metabolic processes [56]. Hypoxia, through HIF-1α, is known to promote tumor progression and immune evasion, contributing to chemoresistance in pancreatic cancer [57,58,59]. Targeting HIF-1α could potentially restore sensitivity to 5-FU in resistant tumors, as shown in other studies [57,60], emphasizing its role in therapy resistance and highlighting ASCs as potential contributors to this resistance mechanism.

The protein array analysis results offer important insights into the adaptive responses of ASCs to the chemotherapy agents OXP and 5-FU. These findings demonstrate how ASCs alter their secretome in response to chemotherapy, potentially contributing to tumor chemoresistance in PDAC (Figure 7).

The cytokine array analysis reveals a significant reduction in pro-angiogenic factors, including VEGF, angiogenin, and angiopoietin-2, with OXP demonstrating a more pronounced suppressive effect compared with 5-FU treatment. While this reduction might initially imply impaired tumor vascularization, it more likely reflects a functional adaptation in ASCs rather than a straightforward inhibition of angiogenesis. This adaptation involves compensatory mechanisms such as the increased expression of stress-related proteins like cystatin C and ECM modulators like EMMPRIN, which support ECM remodeling and promote cellular survival [61,62].

The immune-modulatory and pro-inflammatory responses observed in ASCs further highlight their potential role in fostering chemoresistance. Elevated levels of GROα, GM-CSF, FGF-19, and IL-4 protein levels under OXP treatment suggest that ASCs actively modify the immune landscape of the TME [63]. GROα and GM-CSF are known to recruit immune cells, including myeloid-derived suppressor cells (MDSCs), which can inhibit anti-tumor immunity and support tumor survival [63,64]. Interestingly, IL-4, a cytokine associated with immune suppression such as the polarization of macrophages to a pro-tumoral M2 phenotype, was significantly induced in OXP-treated ASCs but remained almost undetectable in 5-FU-treated cells. Induction of IL-4 under OXP indicates a shift toward an immunosuppressive environment, which may contribute to immune evasion by tumor cells [64,65]. In terms of ECM remodeling, which is critical for tumor invasion and metastasis, ASCs treated with OXP showed a marked increase in the secretion of EMMPRIN and DPPIV, two key cytokines involved in ECM degradation and remodeling [66]. In contrast, 5-FU treatment induced only minor changes in these cytokines, indicating a limited effect on ECM turnover. Furthermore, thrombospondin-1, a cytokine that regulates cell adhesion and migration, was significantly suppressed by 5-FU, while OXP maintained or slightly increased its expression. These results suggest that OXP-treated ASCs may play a more active role in ECM remodeling, potentially facilitating the metastatic cascade, while 5-FU-treated cells exhibit a reduced capacity to promote ECM-dependent processes [67]. The differential regulation of chemokines such as IP-10 (CXCL10) and MIP-3α between OXP and 5-FU treatments underscores the complex and context-specific responses of ASCs. Under OXP treatment, the upregulation of IP-10 may contribute to chemoresistance through mechanisms that recruit immune-suppressive cells, such as MDSCs, which inhibit anti-tumor immune responses and support tumor survival [66,67]. In contrast, uPAR, a cytokine that facilitates tumor cell migration and invasion, was significantly suppressed in ASCs treated with 5-FU but only mildly reduced in OXP-treated cells [68,69]. Similarly, SDF-1α, a chemoattractant that directs cancer cells to metastatic niches, was decreased by both treatments, with 5-FU demonstrating a more pronounced effect [70,71]. The cytokine-secreted profile demonstrates that 5-FU-treated ASCs have a less substantial impact on metastasis-related processes than OXP. These findings align with the low cytotoxicity of 5-FU and the fewer DEGs observed in the RNA-seq data, indicating only a mild induction of cellular stress and adaptive responses in treated ASCs.

These findings suggest that ASCs play a dynamic role in modulating the TME under chemotherapeutic stress. By altering their secretome, ASCs may promote tumor survival through immune evasion, ECM remodeling, and adaptation to angiogenic suppression via hypoxia-related pathways. Future studies should focus on identifying the factors secreted by ASCs in response to chemotherapy and understanding how these factors interact with tumor cells and immune cells. These interactions may significantly influence chemoresistance and impact the overall effectiveness of cancer treatments.

While this study offers valuable insights into the cytotoxic effects of chemotherapeutic agents on PDAC cells and ASCs, several limitations should be acknowledged. For instance, the study was conducted in vitro and may not fully reflect the complexities of in vivo drug responses, where factors such as immune interactions and drug metabolism can influence outcomes. Moreover, focusing solely on the Capan-1 cell line may not capture the diversity of pancreatic cancer subtypes, which are known to exhibit varying drug sensitivities. To address these gaps, future research should include a broader range of cell lines as well as both in vitro co-culture and in vivo models to provide a more comprehensive understanding of drug responses and resistance mechanisms. Additionally, limiting the study to 24 h and 48 h time points may have overlooked longer-term effects and the evolution of resistance. Finally, financial constraints restricted RNA sequencing to select doses and time points, further limiting the scope of the investigation.

## 4. Materials and Methods

### 4.1. Cell Lines and Tissue Culture

#### 4.1.1. Capan-1 Cell Culture

The Capan-1 cell line, derived from human pancreatic ductal adenocarcinoma and obtained from the American Type Culture Collection, was cultured in RPMI 1640 medium supplemented with 15% fetal bovine serum (FBS), 1% antibiotic-antimycotic (AB/AM), and 1% L-glutamine, (all from Biosera, Nuaille, France). The cells were subcultured up to passage 30–35 and maintained in a humidified incubator with 5% CO_2_ at 37 °C. Upon reaching 70–80% confluency, cells were detached using trypsin/EDTA and seeded into 96-well plates for cytotoxicity evaluation of 5-FU and oxaliplatin through the MTT assay.

#### 4.1.2. Adipose-Derived Mesenchymal Stem Cells (ASCs) Isolation and Culture

Adipose tissue collection strictly adhered to the principles outlined in the Helsinki Declaration and received official approval from both the National Public Health and Medical Officer Service (NPHMOS) and the National Medical Research Council (approval numbers: 16821-6/2017/EÜIG, STEM-01/2017). This ensured compliance with EU Member States’ Directive 2004/23/EC, which requires written consent for tissue acquisition. The abdominal tissue samples were obtained as leftovers from the Plastic Surgery—Inpatient Care Unit, Department of Dermatology and Allergology, University of Szeged. Following established protocols, the abdominal adipose tissues were isolated within one hour of plastic surgery [43,44]. The cohort consisted of two female and one male donors, averaging 50.2 years (± 11.7 years). The ASCs were cultured in T25 cm^2^ flasks in a humidified incubator with 5% CO_2_ at 37 °C and were fed every other day with DMEM-high glucose medium (Biosera, Nuaille, France), supplemented with 10% FBS, 1% L-glutamine, and 1% AB/AM Solution (all from Biosera, Nuaille, France). The cells were subcultured up to passages 3–5. Upon reaching 70–80% confluency, the cells were trypsinized and seeded into 96-well plates for cytotoxicity evaluation of 5-FU and oxaliplatin through the MTT assay.

#### 4.1.3. Viability and Metabolic Assay

A viability assay of ASCs was performed using Trypan Blue and an automated cell counter. Cultured cells were trypsinized, pelleted, and resuspended into 2 mL of αMEM. Each cell suspension was diluted 1:1 with Trypan Blue (T8154, Sigma-Aldrich, Budapest, Hungary), and live (unstained) and dead (stained) cells were counted using a hemocytometer. Cell viability was calculated by dividing the number of live cells by the total number of cells.

### 4.2. Validation of Adipose Tissue-Derived Mesenchymal Stem Cell Differentiation Potential into Adipocytes, Chondrocytes, and Osteocytes

To evaluate the differentiation potential of the isolated ASCs, we induced their differentiation into adipocytes, chondrocytes, and osteocytes in vitro [68,69]. The cells were seeded in a 24-well plate at a density of 5 × 10^4^ cells per well. After 24 h, the culture medium was replaced with differentiation medium using Gibco’s StemPro^®^ Adipogenesis, Osteogenesis, and Chondrogenesis Differentiation Kits (Gibco, Thermo Fisher Scientific, Waltham, MA, USA), according to the manufacturer’s instructions. After 21 days of culturing under these conditions, the cells were fixed with 4% methanol-free formaldehyde (Molar Chemicals, Budapest, Hungary) for 20 min at room temperature (RT) in preparation for future staining to assess adipogenic, chondrogenic, and osteogenic differentiation. Nile red staining (Sigma-Aldrich, Merck KGaA, Darmstadt, Germany) was applied to visualize lipid-laden droplets, indicating adipocyte formation. Alizarin red staining (Sigma-Aldrich, Merck KGaA, Darmstadt, Germany) was used to detect mineral deposits associated with osteogenesis, and Toluidine blue staining (Sigma-Aldrich, Merck KGaA, Darmstadt, Germany) was applied to label chondrogenic masses.

### 4.3. Flow Cytometry

We performed surface antigen expression analysis using three-color flow cytometry to confirm that the isolated cells were mesenchymal stem cells. As detailed in our previous studies, we used fluorochrome-labeled antibodies targeting mesenchymal stem cell markers and matching isotype controls [72,73]. Fluorescent signals were measured with the ACEA NovoCyte 2060 flow cytometer and NovoSampler Pro Combo (Agilent Technologies Inc., Santa Clara, CA, USA) (Table 4).

### 4.4. MTT Cell Proliferation Assay

The half-maximal inhibitory concentration (IC50) was evaluated using a metabolic dimethyl thiazolyl diphenyl tetrazolium salt (MTT) assay, which detects cell viability by converting MTT into formazan, a reaction driven by mitochondrial enzymes. In this study, Capan-1 cells (1 × 10^4^ cells per well) and ASCs cells (5 × 10^3^ cells per well) were seeded into 96-well plates and allowed to adhere for 24 h. Cells were subsequently treated with various anticancer drugs, including serial dilutions of 5-fluorouracil (5-FU, F6627) and oxaliplatin (OXP, PHR1525) obtained from Sigma (Sigma-Aldrich, San Diego, CA, USA) at concentrations ranging from 6.25 to 1000 µM. A 5-FU solution (Accord Healthcare, Cork, Ireland) in mg/mL concentration provided by the hospital was also used. Each drug was tested separately to assess its specific effect on cell viability, and no combination treatments were used in this study. Cell proliferation was measured 24 h and 48 h post-treatment for Capan-1 cells and 48 h post-treatment for ASCs. The MTT assay was conducted using the Cell Proliferation Kit I (MTT) (Roche Diagnostics GmbH, Mannheim, Germany), and absorbance was measured at 575 nm using a Synergy HT plate reader (Biotek, Winooski, VT, USA). IC50 values were calculated using GraphPad Prism version 8.0.2 software (GraphPad Software, San Diego, CA, USA). Mitomycin (MTC) at a concentration of 20 μg/mL was used as a positive control in all MTT assays.

### 4.5. Treatment of ASCs for RNA-Seq and Protein Array

Isolated ASCs from three different donors were cultured (2.8 × 10^5^) cells in a T75 cm^2^ flask in a complete DMEM medium and maintained for 24 h. The medium was replaced, and the cells were treated individually with 200 µM 5-FU and 100 µM OXP. Cells were incubated for 48 h under standard conditions (37 °C, 5% CO_2_), with untreated cells used as the control. After 48 h of treatment, the cells were collected for RNA isolation and the supernatant for protein array.

### 4.6. RNA Isolation for RNA Sequencing

Cell pellets were first resuspended in 1 mL of TRI Reagent^®^ (Genbiotech Argentina, Buenos Aires, Argentina) and stored at −80 °C for 24 h. After thawing, 200 µL of chloroform was added to each sample, followed by thorough mixing and incubation at room temperature (RT) for 10 min. The samples were centrifuged at 13,400× *g* for 20 min at 4 °C to allow phase separation. The aqueous phase was carefully transferred to new, clean tubes, and 500 µL of 2-propanol was added and mixed thoroughly, repeating the incubation and phase-separation processes. The supernatants were discarded, and the pellets were washed with 750 µL of 75% EtOH-DEPC. The samples were centrifuged at 7500× *g* for 5 min at 4 °C. After discarding the supernatants, the pellets were dried at 45 °C for 20 min. The dried pellets were then reconstituted in RNase-free water and incubated at 55 °C for 10 min. RNA concentrations were measured using the IMPLEN N50 UV-Vis Nanophotometer (Implen GmbH, Munich, Germany). Finally, the RNA samples were stored at −80 °C until further use.

### 4.7. RNA Sequencing

High-throughput mRNA sequencing was performed using the Illumina platform to generate comprehensive transcriptome data. The quality of total RNA samples was assessed with the Agilent BioAnalyzer, following the manufacturer’s protocol and using the Eukaryotic Total RNA Nano Kit. Only samples with an RNA integrity number (RIN) greater than seven were considered suitable for library preparation. For RNA-Seq library creation, the Ultra II RNA Sample Prep kit (New England BioLabs, Ipswich, MA, USA) was used according to the manufacturer’s instructions. In summary, poly-A RNAs were selectively captured with oligo-dT-conjugated magnetic beads, followed by elution and fragmentation of mRNAs at 94 °C. First-strand cDNA synthesis was performed using random priming reverse transcription, and after second-strand synthesis, double-stranded cDNA was generated. The cDNA fragments underwent end repair, A-tailing, and adapter ligation, and the adapter-ligated fragments were amplified via enrichment PCR to produce sequencing libraries. The sequencing was conducted on the Illumina NextSeq 500 instrument utilizing a single-end 75-cycle sequencing approach.

### 4.8. Protein Array

Supernatants were collected from three different ASC donors, treated with OXP and 5-FU individually, as previously described for RNA sequencing, and stored at −80 °C until further use. After thawing, the supernatants were pooled according to treatment type and analyzed using the Human XL Cytokine Array Kit Proteome Profiler (R&D Systems, Biotechne, McKinley Place NE, Minneapolis, MN, USA) to assess 105 simultaneously secreted cytokines. The array was performed following the manufacturer’s instructions. Arbitrary values of cytokine abundance were calculated as integrated densities, which were measured using the Fiji (ImageJ version 1.53s, NIH, Bethesda, MD, USA) software with Protein Array Analyzer for ImageJ macro by Gilles Carpentier. A comprehensive statistical analysis was performed for each cytokine to evaluate the differences in cytokine levels between the treatment groups. Changes were considered significant when there was a doubling (fold change = 2, Log2FC = 1) between untreated (CTRL) and treated cells (5-FU and OXP), or if there was a twofold decrease (factor change = 0.5, Log2FC = −1) between the groups. Initially, a Welch’s t-test was conducted to compare the mean values of the two groups assuming unequal variances. This test is robust to group size and variance differences, and a *p*-value < 0.05 was considered statistically significant. In parallel, a Mann–Whitney U-test, a nonparametric alternative to the *t*-test, was applied to compare the median values between the groups. This test is particularly useful for data that do not follow a normal distribution, and a *p*-value < 0.05 was also used as the threshold for significance. Additionally, a Kruskal–Wallis test, a nonparametric analysis of variance (ANOVA), was used to assess differences between the groups. If this test indicated statistical significance (*p*-value < 0.05), a post hoc Dunn test was performed to identify significant pairwise comparisons between the groups. The Dunn test results were corrected for multiple comparisons using the Bonferroni adjustment to minimize the risk of type I errors. All statistical computations were carried out using R (version 4.4.2, Auckland, New Zealand).

### 4.9. Data Analysis

Gene expression analysis was conducted in R (version 4.2.0, Auckland, New Zealand) with a pre-filtering step to remove genes with low expression, specifically those with a total count of less than ten across all samples. We utilized principal component analysis (PCA) to represent sample distances using the PCA tools package visually, and notably, it did not reveal any significant batch effects. Differential expression analysis was executed using DESeq2. Significantly differentially expressed genes (DEGs) were identified based on adjusted *p*-values below 0.05 and a log2-fold change threshold set at 0. Heatmaps were generated using the R package Complex Heatmap to visualize these DEGs. Pearson correlation was applied to rows and columns, and z-scores were calculated from normalized count data. The normalization process employed DESeq2 counts (dds, normalized = T). Additionally, volcano plots were generated using the Enhanced Volcano package. In the gene set enrichment analysis (GSEA) context, DEGs were ordered based on their log2-fold changes and utilized as input. For GSEA, we leveraged the R package Cluster Profiler with an adjusted *p*-value cutoff of 0.05 for Gene Ontology (GO) and Kyoto Encyclopedia of Genes and Genomes (KEGG) GSEA. Furthermore, GO terms were clustered further using rrvgo with default settings, and heatmaps representing various pathways were created using pre-selected gene sets. The DEGs in each gene set were consistent with the approach used for the heatmap visualization of all genes. DEGs were selected and ordered according to their log2-fold changes. This ordered list was then employed as input for the gene set enrichment analysis (GSEA), which aligned with the Gene Ontology and KEGG databases. Additionally, pathway analysis was performed using the Hallmark gene set system from the Molecular Signatures Database (MSigDB), which includes curated gene sets representing vital biological processes (BPs) such as cell cycle regulation, metabolism, and immune responses. This approach was used to identify significantly enriched pathways from the DEGs data of ASCs treated with 5-FU and OXP.

### 4.10. Statistical Analysis

Statistical analyses were conducted using GraphPad Prism 8.0.2 (GraphPad Software, San Diego, CA, USA) with Student’s unpaired *t*-tests to compare means. The data are presented as mean values with standard deviations, and statistical significance was set at *p*-value < 0.05. Furthermore, the nonparametric Mann–Whitney U test and ANOVA with multiple comparisons of the HSD–Tukey test were utilized to identify any additional statistically significant differences.

## 5. Conclusions

This study highlights the potential role of ASCs in pancreatic cancer chemoresistance, particularly in response to OXP and 5-FU treatments. The findings reveal distinct adaptive responses in ASCs, characterized by differential transcriptional reprogramming and cytokine secretion profiles, which dynamically reshape the TME. OXP induces extensive transcriptional changes, upregulating stress response pathways, immune-modulatory cytokines, and ECM remodeling factors, thereby fostering tumor survival and metastasis. Conversely, 5-FU elicits a milder impact on ASCs, resulting in moderate reductions in pro-tumoral cytokines, limited ECM remodeling, and reduced immune modulation.

The resilience of ASCs to chemotherapeutic agents underscores their potential role in shielding tumor cells from treatment-induced stress and maintaining TME homeostasis. The interplay between ASC-mediated immune evasion, hypoxia-driven survival pathways, and ECM remodeling highlights key mechanisms of chemoresistance, emphasizing the importance of targeting stromal components in cancer therapy.

Our findings suggest that therapeutic strategies combining direct tumor targeting with modulation of ASC-secreted factors—such as GROα, GM-CSF, EMMPRIN, or IL-4—may enhance treatment efficacy. Targeting hypoxia signaling, immune-modulatory pathways, or ECM remodeling could mitigate the supportive role of ASCs in the TME and reduce chemoresistance.

Future research should focus on dissecting the precise roles of ASC-derived factors in tumor progression and resistance, leveraging multi-omics approaches to identify novel therapeutic targets. A deeper understanding of the stromal–tumor crosstalk will be crucial for developing innovative strategies to overcome resistance and improve outcomes for patients with PDAC and other stromal-rich tumors.

## Figures and Tables

**Table 1:** GO enrichment analysis of DEGs in ASCs treated with OXP and 5-FU.

Positively Enriched GO Terms							
GO ID	Description	Bg Ratio	*p*-Value	*p*. Adjust	q-Value	Gene ID	Count
0006977	DNA damage response, signal transduction by p53 class mediator resulting in cell cycle arrest	18/18,903	0.000101960656047455	0.0253341548649552	0.0162517934743993	MDM2/PIDD1	2
0010948	Negative regulation of cell cycle process	318/18,903	0.000121909527970945	0.0253341548649552	0.0162517934743993	BCL6/MDM2/PIDD1/RBM14	4
0046599	Regulation of centriole replication	23/18,903	0.000168185906455739	0.0253341548649552	0.0162517934743993	CEP295NL/RBM14	2
0071456	Cellular response to hypoxia	151/18,903	0.000259259235342849	0.0253341548649552	0.0162517934743993	MDM2/STC1/TIGAR	3
0031571	Mitotic G1 DNA damage checkpoint signaling	29/18,903	0.000269096926508324	0.0253341548649552	0.0162517934743993	MDM2/PIDD1	2
0044819	Mitotic G1/S transition checkpoint signaling	30/18,903	0.000288175849364675	0.0253341548649552	0.0162517934743993	MDM2/PIDD1	2
0036294	Cellular response to decreased oxygen levels	159/18,903	0.000301744137941842	0.0253341548649552	0.0162517934743993	MDM2/STC1/TIGAR	3
0045786	Negative regulation of cell cycle	405/18,903	0.000308014040911309	0.0253341548649552	0.0162517934743993	BCL6/MDM2/PIDD1/RBM14	4
0072331	Signal transduction by p53 class mediator	173/18,903	0.000386468887239921	0.0262999193876633	0.0168713288665062	EDA2R/MDM2/PIDD1	3
0071453	Cellular response to oxygen levels	175/18,903	0.00039969482352072	0.0262999193876633	0.0168713288665062	MDM2/STC1/TIGAR	3
0007099	Centriole replication	42/18,903	0.000567021211169682	0.0339181779045137	0.0217584215960806	CEP295NL/RBM14	2
0098534	Centriole assembly	46/18,903	0.000680266788329752	0.0359445339843582	0.0230583236725766	CEP295NL/RBM14	2
0010824	Regulation of centrosome duplication	47/18,903	0.000710150367472122	0.0359445339843582	0.0230583236725766	CEP295NL/RBM14	2
0046605	Regulation of centrosome cycle	54/18,903	0.000936837607682431	0.0440313675610742	0.0282460060662146	CEP295NL/RBM14	2
0010332	Response to gamma radiation	57/18,903	0.00104331346356804	0.0442341420950184	0.0283760853945007	MDM2/TIGAR	2
0045930	Negative regulation of mitotic cell cycle	246/18,903	0.00107560223939255	0.0442341420950184	0.0283760853945007	BCL6/MDM2/PIDD1	3
0043122	Regulation of I-kappaB kinase/NF-kappaB signaling	255/18,903	0.00119298433051044	0.0461755111456394	0.0296214685160797	EDA2R/PIDD1/TNFRSF10B	3
**Negatively Enriched GO Terms**							
**GO ID**	**Description**	**Bg Ratio**	***p*** **-Value**	***p*** **. Adjust**	**q-Value**	**Gene ID**	**Count**
0000280	Nuclear division	481/18,903	1.76881066719348 × 10^−18^	1.47872571777375 × 10^−15^	1.03894352873049 × 10^−15^	AURKA/BUB1/BUB1B/CCNB1/CCNB2/CENPE/CENPF/DLGAP5/KIF11/KIF20B/KIF23/NDC80/NUF2/NUSAP1/RAD51/TOP2A/TTK	17
0007088	Regulation of mitotic nuclear division	118/18,903	1.28092889781954 × 10^−16^	4.8902786348109 × 10^−14^	3.43587947014396686 × 10^−14^	AURKA/BUB1/BUB1B/CCNB1/CENPF/DLGAP5/KIF20B/NDC80/NUF2/NUSAP1/TTK	11
0000819	Sister chromatid segregation	239/18,903	1.75488467756372 × 10^−16^	4.8902786348109 × 10^−14^	3.43587947396686 × 10^−14^	BUB1/BUB1B/CCNB1/CENPE/CENPF/DLGAP5/KIF11/KIF23/NDC80/NUF2/NUSAP1/TOP2A/TTK	13
0140014	Mitotic nuclear division	325/18,903	2.50022223332157 × 10^−16^	5.22546446764208 × 10^−14^	3.67137896366693 × 10^−14^	AURKA/BUB1/BUB1B/CCNB1/CENPE/CENPF/DLGAP5/KIF11/KIF20B/KIF23/NDC80/NUF2/NUSAP1/TTK	14
0000070	Mitotic sister chromatid segregation	204/18,903	1.20987483051498 × 10^−15^	1.82830384900337 × 10^−13^	1.28455495812627 × 10^−13^	BUB1/BUB1B/CCNB1/CENPE/CENPF/DLGAP5/KIF11/KIF23/NDC80/NUF2/NUSAP1/TTK	12
0051783	Regulation of nuclear division	145/18,903	1.31217979593543 × 10^−15^	1.82830384900337 × 10^−13^	1.28455495812627 × 10^−13^	AURKA/BUB1/BUB1B/CCNB1/CENPF/DLGAP5/KIF20B/NDC80/NUF2/NUSAP1/TTK	11
0044772	Mitotic cell cycle phase transition	473/18,903	1.55567542073125 × 10^−15^	1.85792093104475 × 10^−13^	1.30536373649329 × 10^−13^	AURKA/BRCA1/BUB1/BUB1B/CCNA2/CCNB1/CCNB2/CENPE/CENPF/CIT/DLGAP5/MELK/NDC80/NUF2/TTK	15
0007059	Chromosome segregation	382/18,903	2.36061743337282 × 10^−15^	2.4668452178746 × 10^−13^	1.73319016818689 × 10^−13^	BRCA1/BUB1/BUB1B/CCNB1/CENPE/CENPF/DLGAP5/KIF11/KIF23/NDC80/NUF2/NUSAP1/TOP2A/TTK	14
0098813	Nuclear chromosome segregation	321/18,903	8.09097373728085 × 10^−15^	7.51561560485199 × 10^−13^	5.28042496538329 × 10^−13^	BUB1/BUB1B/CCNB1/CENPE/CENPF/DLGAP5/KIF11/KIF23/NDC80/NUF2/NUSAP1/TOP2A/TTK	13
0051304	Chromosome separation	135/18,903	3.88243219597149 × 10^−14^	3.24571331583216 × 10^−12^	2.28041806879167 × 10^−12^	BUB1/BUB1B/CCNB1/CENPE/CENPF/DLGAP5/NDC80/NUF2/TOP2A/TTK	10

**Table 2:** GSEA results from the Hallmark, KEGG, and GO Pathway databases for ASCs treated with OXP.

Hallmark Gene Sets of ASDMSCs Treated by OXP							
	Pathway	*p*-Value	*p*-Adj	log2err	ES	NES	Size
1	HALLMARK_E2F_TARGETS	2.64139101652653 × 10^−5^	0.000220115918043877	0.575610261071129	−0.340157293053717	−2.19499534337419	66
2	HALLMARK_PROTEIN_SECRETION	5.91563187836908 × 10^−5^	0.00042254513416922	0.557332238758646	−0.37601381727437	−2.23266725677663	48
3	HALLMARK_TGF_BETA_SIGNALING	0.000218576664884453	0.00136610415552783	0.518848077743792	−0.495322402827272	−2.25846306096561	22
4	HALLMARK_WNT_BETA_CATENIN_SIGNALING	0.000590324063336932	0.00327957812964962	0.477270815362862	−0.52540555272117	−2.22228354009291	18
5	HALLMARK_GLYCOLYSIS	0.0013175339500311	0.00658766975015548	0.45505986738723	−0.263389231583071	−1.72899685956862	71
6	HALLMARK_APICAL_SURFACE	0.0017804176406084	0.00809280745731091	0.45505986738723	−0.491393827829415	−2.04389947310002	17
7	HALLMARK_ANDROGEN_RESPONSE	0.0026784748393661	0.0111603118306921	0.431707695803346	−0.310678503446223	−1.77233833738595	43
8	HALLMARK_IL6_JAK_STAT3_SIGNALING	0.00834837545126354	0.0321091363510136	0.167658528065765	0.503377946262252	1.68855652293838	31
9	HALLMARK_INTERFERON_GAMMA_RESPONSE	0.0132659507264687	0.047378395451674	0.12797030576243	0.423017595767414	1.56993531942059	57
10	HALLMARK_OXIDATIVE_PHOSPHORYLATION	0.0169363189037552	0.0564543963458505	0.352487857583619	0.190907632331622	1.40892610341346	116
**KEGG Pathway Gene Sets of ASCs Treated by OXP**							
	**Pathway**	***p*** **-Value**	***p*** **-Adj**	**log2err**	**ES**	**NES**	**Size**
1	KEGG_CYTOKINE_CYTOKINE_RECEPTOR_INTERACTION	3.93732090514141 × 10^−7^	0.0000346492769444532	0.674962860011025	0.532331938126544	2.05892651987083	80
2	KEGG_PATHWAYS_IN_CANCER	4.22552157859185 × 10^−7^	0.0000346492769444532	0.674962860011025	−0.294701843495029	−2.15756612113785	130
3	KEGG_WNT_SIGNALING_PATHWAY	7.93012559407585 × 10^−7^	0.0000433513532476146	0.659444398037935	−0.393751027771448	−2.47883362699721	63
4	KEGG_RIBOSOME	1.8006005614366 × 10^−5^	0.000738246230189004	0.575610261071129	0.508195995347252	1.95272043832977	74
5	KEGG_CELL_CYCLE	6.56683118791417 × 10^−5^	0.00215392062963585	0.538434096309916	−0.368250635338693	−2.17428371995909	49
6	KEGG_COLORECTAL_CANCER	0.000153127064422625	0.00418547309421842	0.518848077743792	−0.424324314956772	−2.2464797217589	34
7	KEGG_ADHERENS_JUNCTION	0.000344717035697592	0.00471113282120042	0.49849310876659	−0.417114186960923	−2.19630436832298	33
8	KEGG_PROPANOATE_METABOLISM	0.000254144405157146	0.00471113282120042	0.49849310876659	−0.587093416395374	−2.31891722502165	15
9	KEGG_REGULATION_OF_ACTIN_CYTOSKELETON	0.000223272560199641	0.00471113282120042	0.518848077743792	−0.289865264978343	−1.93135681744662	75
10	KEGG_SYSTEMIC_LUPUS_ERYTHEMATOSUS	0.000325945507623177	0.00471113282120042	0.49849310876659	0.624911870842555	2.01016409806685	25
**GO Pathway Gene Sets of ASCs Treated by OXP**							
	**Pathway**	***p*** **-Value**	***p*** **-Adj**	**log2err**	**ES**	**NES**	**Size**
1	GOCC_CILIARY_BASAL_BODY	7.82720516782125 × 10^−7^	0.0000919926819135698	0.659444398037935	−0.39610119737703	−2.44040401476571	60
2	GOMF_CYTOKINE_RECEPTOR_BINDING	1.51281379650273 × 10^−9^	0.000000906780589623734	0.788186810800237	0.582963622296549	2.24926964536283	80
3	GOMF_PHOSPHORIC_ESTER_HYDROLASE_ACTIVITY	1.3092422431839 × 10^−9^	0.000000871955333960478	0.788186810800237	−0.333513038562531	−2.41942991352463	126
4	GOBP_REGULATION_OF_PROTEIN_SERINE_THREONINE_KINASE_ACTIVITY	7.01033059960302 × 10^−7^	0.000084039843228041	0.659444398037935	−0.257783461836265	−1.98310722278176	152
5	GOBP_CELLULAR_RESPONSE_TO_ORGANIC_CYCLIC_COMPOUND	6.54759701956862 × 10^−7^	0.0000800944827250904	0.659444398037935	−0.237847374889051	−2.05107083041299	201
6	GOMF_PHOSPHATASE_ACTIVITY	2.66353767958835 × 10^−8^	0.0000079826224257263	0.73376198835648	−0.352246801743327	−2.49094501452385	101
7	GOBP_CILIUM_ORGANIZATION	6.36062716975486 × 10^−7^	0.0000794283317823138	0.659444398037935	−0.275699356833308	−2.05428254765	141
8	GOBP_HEART_MORPHOGENESIS	6.08811861023075 × 10^−7^	0.0000776429424462194	0.659444398037935	−0.358748271702841	−2.38680754796361	82
9	GOBP_REGIONALIZATION	6.07629933943173 × 10^−7^	0.0000776429424462194	0.659444398037935	−0.319006359814004	−2.2465575880912	103
10	GOBP_PATTERN_SPECIFICATION_PROCESS	1.97618354957955 × 10^−8^	0.00000633035626394535	0.73376198835648	−0.307348031514522	−2.28721000698764	135

**Table 3:** GSEA results from the Hallmark and GO Pathway databases for ASCs treated with 5-FU.

Hallmark Pathway Gene Sets of ASCs Treated by 5-FU							
	Pathway	*p*-Value	*p*-Adj	log2err	ES	NES	Size
1	HALLMARK_G2M_CHECKPOINT	0.0000733471109727229	0.000682362573410719	0.538434096309916	−0.558868261837081	−1.7830668390697	45
2	HALLMARK_E2F_TARGETS	0.000136472514682144	0.000682362573410719	0.518848077743792	−0.574875140341789	−1.80515308646078	38
3	HALLMARK_HYPOXIA	0.000934151233258401	0.00311383744419467	0.477270815362862	0.769430870017944	2.24004089128129	7
4	HALLMARK_MITOTIC_SPINDLE	0.00196565280364163	0.00491413200910408	0.335068558717014	−0.567565074528727	−1.68661253301052	25
5	HALLMARK_SPERMATOGENESIS	0.0238582140422631	0.0477164280845262	0.0986244904449457	−0.61558780552373	−1.52724795175338	10
6	HALLMARK_ESTROGEN_RESPONSE_LATE	0.0531142529614062	0.088523754935677	0.0692037251597512	−0.691973854147228	−1.42865347619181	5
7	HALLMARK_GLYCOLYSIS	0.100535460605398	0.14362208657914	0.0453371931652217	−0.507474988249809	−1.3390527344082	13
8	HALLMARK_P53_PATHWAY	0.605299860529986	0.751921198998004	0.0372972016676791	0.363636363636364	0.891284030826118	5
9	HALLMARK_MYC_TARGETS_V1	0.676729079098204	0.751921198998004	0.0135521440941112	−0.420312088040893	−0.867778922666198	5
10	HALLMARK_MTORC1_SIGNALING	0.874798619102417	0.874798619102417	0.00810455465169815	−0.283125336361726	−0.685983995043019	9
**GO Pathway Gene Sets of ASCs Treated by 5-FU**							
	**Pathway**	***p*** **-Value**	***p*** **-Adj**	**log2err**	**ES**	**NES**	**Size**
1	GOBP_CELL_CYCLE	2.79067384940996 × 10^−11^	1.58231207261545 × 10^−8^	0.863415391606693	−0.685309035511196	−2.27333326581027	97
2	GOBP_CELL_CYCLE_PROCESS	3.82674947408738 × 10^−9^	7.23255650602515 × 10^−7^	0.761460801445585	−0.651897388248435	−2.16371155173349	88
3	GOBP_CELL_DIVISION	1.40683280616485 × 10^−7^	1.13953457299353 × 10^−5^	0.690132458796796	−0.632482709801711	−2.055589722267	56
4	GOBP_CHROMOSOME_ORGANIZATION	4.06803322002345 × 10^−6^	0.000177428833519484	0.610526878385931	−0.603365321057273	−1.94936769929251	52
5	GOBP_CHROMOSOME_SEGREGATION	1.29447171412157 × 10^−8^	1.46793092381386 × 10^−6^	0.747739663149885	−0.68303550124922	−2.18639772376246	46
6	GOBP_CYTOSKELETON_ORGANIZATION	1.58714316917903 × 10^−5^	0.000599940117949674	0.575610261071129	−0.58390599218358	−1.88649801515187	52
7	GOBP_MITOTIC_CELL_CYCLE	2.96260527345182 × 10^−9^	7.23255650602515 × 10^−7^	0.774939030136436	−0.644228764699433	−2.13171387737599	79
8	GOBP_MITOTIC_CELL_CYCLE_PROCESS	8.47450055550495 × 10^−9^	1.20126045374283 × 10^−6^	0.747739663149885	−0.635828913368128	−2.10045721627125	76
9	GOBP_MITOTIC_NUCLEAR_DIVISION	1.12901172508325 × 10^−06^	7.11277386802448 × 10^−5^	0.643551836150722	−0.640638667096561	−2.05068539033824	46
10	GOBP_NUCLEAR_CHROMOSOME_SEGREGATION	1.26791003202673 × 10^−7^	1.13953457299353 × 10^−5^	0.690132458796796	−0.668286487412083	−2.11091785107083	40
11	GOBP_ORGANELLE_FISSION	2.5763252011723 × 10^−7^	1.82597048633087. × 10^−5^	0.674962860011025	−0.632903138174849	−2.05103119716214	54
12	GOBP_REGULATION_OF_CELL_CYCLE	2.5659740132735 x. 10^−6^	0.000132264296866007	0.627256739718528	−0.590061640981375	−1.94396251426521	70
13	GOBP_SISTER_CHROMATID_SEGREGATION	1.53348302367881 × 10^−6^	8.69484874425883 × 10^−5^	0.643551836150722	−0.650394050059615	−2.04361563736296	38
14	GOCC_MICROTUBULE_CYTOSKELETON	4.61134563187446 × 10^−6^	0.000186759498090915	0.610526878385931	−0.592507368359088	−1.92870493250568	57
15	GOCC_SPINDLE	3.52663387478052 × 10^−6^	0.000166633450583379	0.627256739718528	−0.617148091472953	−1.95344908812434	41

**Table 4:** The phenotype of ASCs used in the study.

CD Markers	Mean	SD
HLA-DR	0.9	0.7
CD29	99.0	0.4
CD34	1.9	1.5
CD47	96.1	1.6
CD49A	92.5	5.6
CD51	82.9	5.8
CD73	99.0	0.1
CD90	90.9	1.0
CD105	88.1	6.0
CD166	95.9	0.8

## Data Availability

All data generated and analyzed during this study are included in this manuscript (and its Supplementary Materials).

## Supplementary Materials

The following supporting information can be downloaded at https://www.mdpi.com/article/10.3390/ijms26010390/s1.
