# Supplementary material for: Chemoresistance in Pancreatic Cancer: The Role of Adipose-Derived Mesenchymal Stem Cells and Key Resistance Genes
Source: Int J Mol Sci. 2025 Jan 4;26(1):390. doi: 10.3390/ijms26010390 (PMC11720846; doi:10.3390/ijms26010390)
Supplement: Supplementary file 1 [file ijms-26-00390-s001.zip › ijms-3260380-supplementary figures and table.pdf]

ShahramP_5FUMSC_ Supplementary figures and table_R1

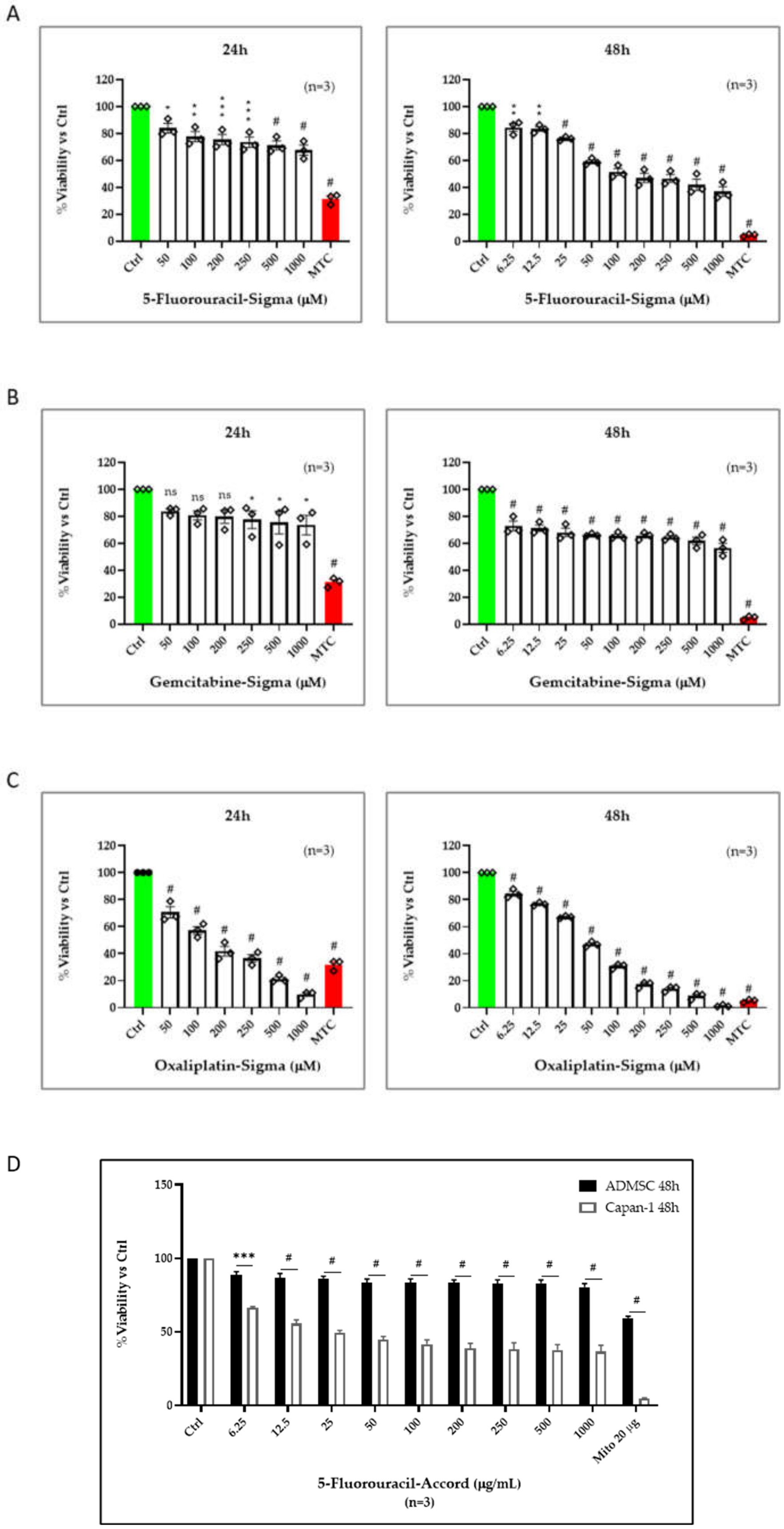

Figure S1. The results of Cytotoxicity effect of anticancer agents on Capan-1 cells at 24- and 48-hours post-treatment with concentrations 6.25- 1000 µM of **A)** 5-Fluorouracil (5-FU) **B)** Gemcitabine (GEM), and **C)** Oxaliplatin (OXP), on the Capan-1 cell line. MTC: Mitomycin 20 µM/mL was used as a positive control. (mean ± SD, n = 3). **D)** Antiproliferative effect of 5-Fluorouracil (Accord), which has been used for patients in the hospital on Capan-1 and ASC after 48 hours post-treatment. [Drug concentration, µg/mL] (mean ± SD, n = 3).

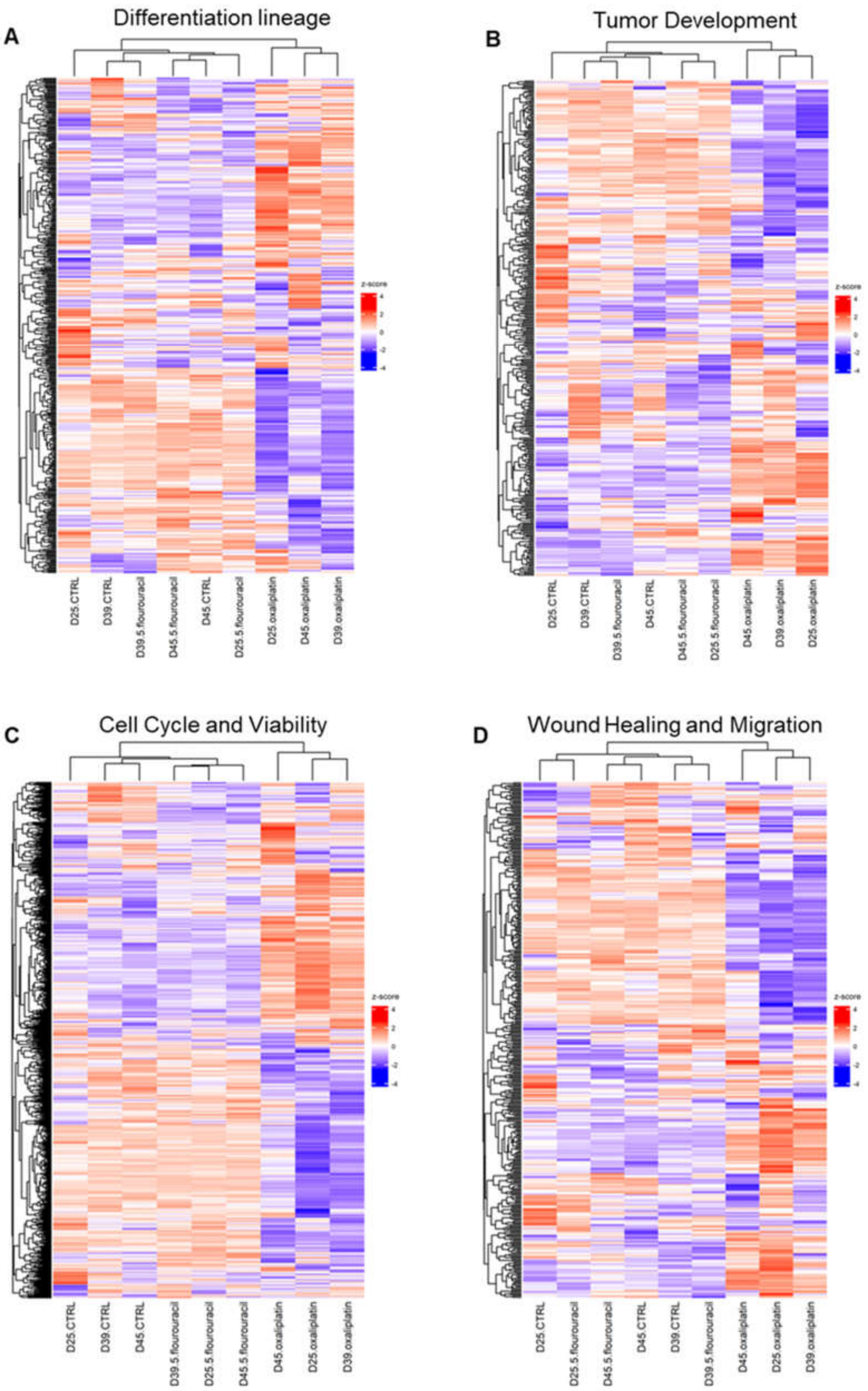

Figure S2. Heat maps display the differentially expressed genes (DEGs) in the following pathways for ASCs treated with oxaliplatin and 5-fluorouracil compared to the control: A) differentiation lineage, B) tumor development, C) cell cycle-viability , and D) wound healing and migration. Z-scores were calculated from count data transformed using DESeq2's variance stabilizing transformation. Significantly upregulated genes are shown in orange, while significantly downregulated genes are highlighted in blue. D25, D39, and D45 represent three biological donor codes (n=3).

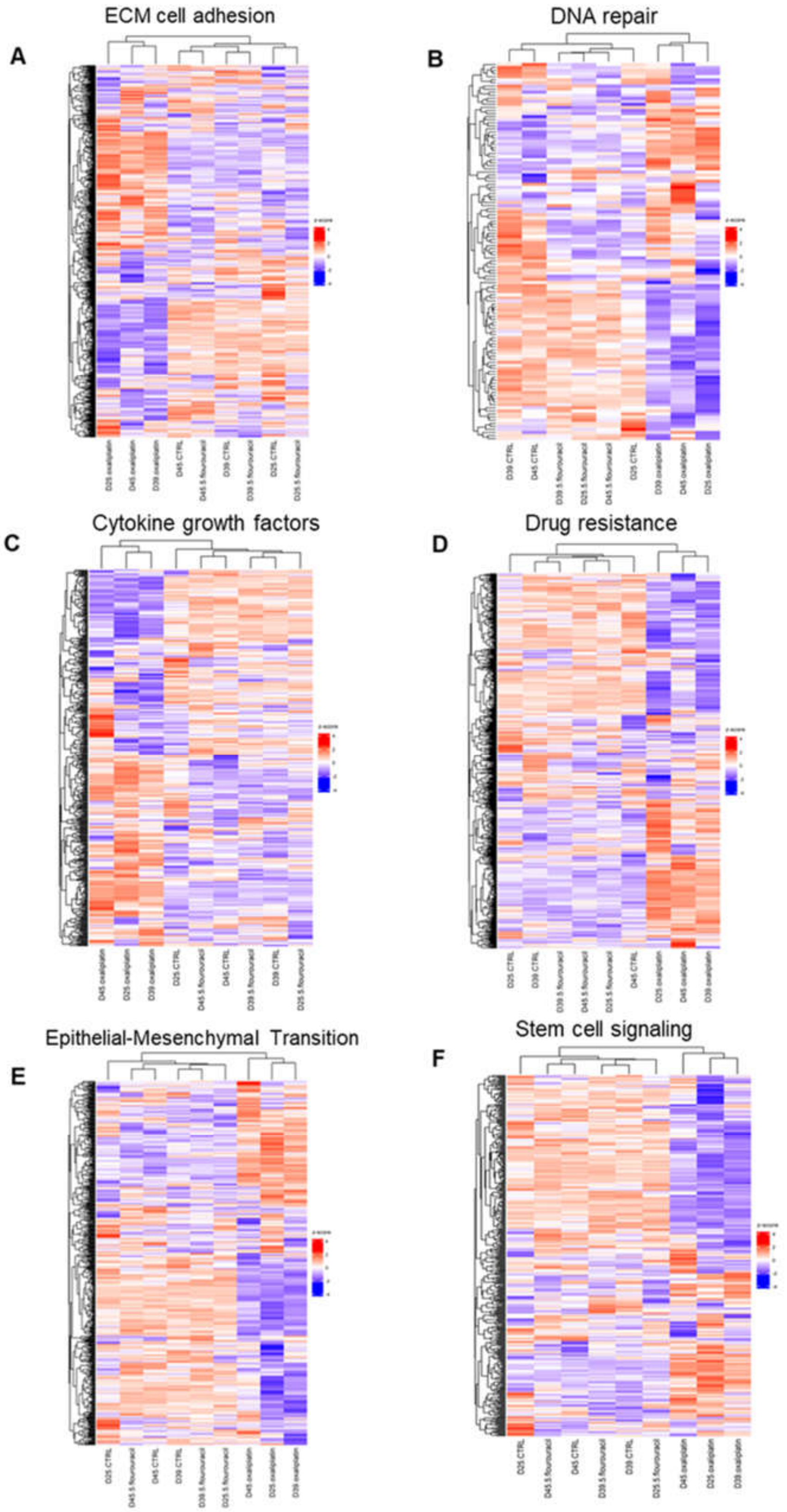

Figure S3. Heat maps represent the differentially expressed genes (DEGs) in the following pathways for ASCs treated with OXP, 5-FU, and untreated control: A) extracellular matrix (ECM) cell adhesion, B)DNA repair, C) Cytokine growth factors, D) Drug-resistant, E) Epithelial-mesenchymal transition, and F) Stem cell signaling. Z-scores were calculated from count data transformed using DESeq2's variance stabilizing transformation. Significantly upregulated genes are shown in orange, while significantly downregulated genes are highlighted in blue. D25, D39, and D45 represent three biological donor codes (n=3).

Table S1. Results of IC50 values for Capan-1 at 24 and 48 hours and ASCs at 48 hours post-treatment with oxaliplatin (OXP), gemcitabine (GEM), and 5-fluorouracil (5-FU). **ns:** Non-significant

		IC50% (μM)		
**Cell line**	**Time exposure** (hour)	**OXP**	**GEM**	**5-FU**
Capan-1	**24**	140±10	ns	ns
	**48**	50±5	1000±150	200±15
AD-MSC	**48**	250±25	ns	ns
