# Supplementary material for: Chemoresistance in Pancreatic Cancer: The Role of Adipose-Derived Mesenchymal Stem Cells and Key Resistance Genes
Source: Int J Mol Sci. 2025 Jan 4;26(1):390. doi: 10.3390/ijms26010390 (PMC11720846; doi:10.3390/ijms26010390)
Supplement: Supplementary file 1 [file ijms-26-00390-s001.zip › ijms-3260380-supplementary.pdf]

Supplementary information

(*ID*: The GO identifier, *Description*: A brief description of the biological process. *GeneRatio*: The ratio of genes associated with the GO term in the dataset. *BgRatio*: The background ratio of genes related to the GO term. *p-value*: The unadjusted p-value for the enrichment test. p.adjust: The adjusted p-value (after multiple testing correction). q-value: False discovery rate (FDR)-corrected q-value. geneID: Identifiers for the genes associated with the GO term. *Count:* The number of genes associated with the GO term. Columns are as follows: ID: The GO identifier. Description: A description of the biological process involved. GeneRatio: The ratio of genes from the dataset associated with the GO term. BgRatio: The background ratio of genes related to the GO term. p-value: The p-value from the statistical test for enrichment. p.adjust: The p-value after adjustment for multiple comparisons. q-value: FDR-corrected q-value. geneID: The list of genes contributing to the enrichment. Count: The number of genes associated with the term. external_gene_name: The gene name.

ensembl_gene_id: The Ensemble gene identifier.

description: A brief description of the gene function.

entrezgene_id: The Entrez gene identifier.

baseMean_fluorouracil: The mean expression level in the fluorouracil condition.

log2FoldChange_fluorouracil: Log2-fold change in expression for fluorouracil treatment.

lfcSE_fluorouracil: Standard error for the log2-fold change.

pvalue_fluorouracil: The p-value for differential expression under fluorouracil treatment.

padj_fluorouracil: Adjusted p-value for fluorouracil treatment.

baseMean_oxaliplatin: The mean expression level in the oxaliplatin condition.

log2FoldChange_oxaliplatin: Log2-fold change for oxaliplatin treatment.

lfcSE_oxaliplatin: Standard error for the log2-fold change.

pvalue_oxaliplatin: The p-value for differential expression under oxaliplatin treatment.

padj_oxaliplatin: Adjusted p-value for oxaliplatin treatment.
